# What factors affect the carriage of epinephrine auto-injectors by teenagers?

**DOI:** 10.1186/2045-7022-2-3

**Published:** 2012-02-02

**Authors:** Clare Macadam, Julie Barnett, Graham Roberts, Gary Stiefel, Rosemary King, Michel Erlewyn-Lajeunesse, Judith A Holloway, Jane S Lucas

**Affiliations:** 1 Academic Unit of Clinical and Experimental Sciences, Faculty of Medicine, University of Southampton, Southampton, UK; 2 Department of Information Systems and Computing, Brunel University, Uxbridge, UK; 3 University Hospital Southampton Foundation NHS Trust, Southampton, UK

**Keywords:** Food allergy, adolescent, adherence, anaphylaxis, auto-injector, patient education

## Abstract

**Background:**

Teenagers with allergies are at particular risk of severe and fatal reactions, but epinephrine auto-injectors are not always carried as prescribed. We investigated barriers to carriage.

**Methods:**

Patients aged 12-18 years old under a specialist allergy clinic, who had previously been prescribed an auto-injector were invited to participate. Semi-structured interviews explored the factors that positively or negatively impacted on carriage.

**Results:**

Twenty teenagers with food or venom allergies were interviewed. Only two patients had used their auto-injector in the community, although several had been treated for severe reactions in hospital. Most teenagers made complex risk assessments to determine whether to carry the auto-injector. Most but not all decisions were rational and were at least partially informed by knowledge. Factors affecting carriage included location, who else would be present, the attitudes of others and physical features of the auto-injector. Teenagers made frequent risk assessments when deciding whether to carry their auto-injectors, and generally wanted to remain safe. Their decisions were complex, multi-faceted and highly individualised.

**Conclusions:**

Rather than aiming for 100% carriage of auto-injectors, which remains an ambitious ideal, personalised education packages should aim to empower teenagers to make and act upon informed risk assessments.

## Background

Food allergy is common [1], affecting 2.3% of 11 to 15 year olds [2], and evidence suggests that the number of severe food allergic reactions is increasing [3]. In the UK there were 48 deaths from food allergy between 1999 and 2006 [4] with peanut and tree nut allergy accounting for the majority of deaths. Although a previous history of anaphylaxis, asthma and peanut allergy have been identified as risk factors for anaphylaxis, there is no reliable way of predicting who will have a life-threatening reaction. Teenagers are at particular risk with the peak incidence of deaths from anaphylaxis associated with peanut and tree nut allergy occurring in the 15 to 24 age group [4]. First line treatment of anaphylaxis in the community is intramuscular injection of epinephrine (adrenaline) in the thigh [5] via an auto-injector with administration of a second dose if the symptoms persist or deteriorate.

Previous studies in our clinic suggest that teenagers and young adults take risks when managing their allergies [6-10]. They do not always carry their emergency medication, eat foods labelled with "may contain" warnings, and don't tell the people around them about their allergies. Absence of an auto-injector was found to be a common factor in anaphylactic fatalities in USA [11].

Qualitative studies provide the depth of understanding concerning experiences, thoughts and opinions of allergic individuals [12] that is required to inform clinical practice and intervention strategies. An in-depth study has recently provided important insights into the barriers to auto-injector use by teenagers recruited via primary care, a patient support group and a press release [13]; all were considered high risk for anaphylaxis and patients were excluded if their reactions had been in early childhood. The study identified multifaceted reasons that prevent use of the auto-injectors, including failure to recognise the severity of reaction, poor training regarding how to administer the dose, and failure to carry the device. It is imperative that the auto-injector is carried if there is any chance for it to be used. We therefore focused on the attitudes of adolescents under the care of a specialist paediatric allergy clinic that might impact on whether they carry their prescribed auto-injector.

## Methods

Ethical approval was granted by Isle of Wight, Portsmouth and South East Hampshire Research Ethics Committee (LREC10/H0501/31). Informed consent and assent was gained from parent and participant.

### Participant Selection and Recruitment

Consecutive patients between 12 and 18 years old, who had previously been prescribed an auto-injector were interviewed prior to their routine clinic appointment or whilst attending the day ward for immunotherapy at Southampton University Hospitals NHS Trust (SUHT). Decisions to prescribe an auto-injector were made by a paediatric allergist on clinical lines broadly based on EAACI guidelines [14],. Following the guidelines, the majority of prescriptions were based on previous moderate or severe reactions, but some teenagers received prescriptions because of other risk factors, primarily peanut or tree nut allergy in asthmatic patients, and insect venom allergy in patients living remotely from medical care. By 12 years participants will have had at least one training session about anaphylaxis and auto-injectors, aimed at them rather than their parents.

### Questionnaire and Interview

Demographic data and information about their allergic history was obtained in a short questionnaire completed before the interview. The severity of a participant's worst ever reaction to was graded using a classification previously used for peanut allergy [15]. All interviews were conducted by one interviewer trained in qualitative interview techniques. Interviews were conducted face-to-face between the interviewer and teenager in a private clinical area. One interview was conducted at a bedside. The interviews were semi-structured, with a prompt sheet containing areas to be covered, including their allergic experiences, auto-injector carrying practices, and reasons for and against carriage. Interviews were digitally recorded, anonymised and transcribed by an experienced transcriber. Transcripts were checked for accuracy by the interviewer before coding. Thematic coding [16] was used to identify the main themes in the data. The themes capture the main positions that the participants took and include both areas of consensus and difference between participants. The themes and their interpretation were agreed between two of the researchers (CM and JL) and agreed with the wider research team. We had planned 20-30 interviews; there was no significant development of the themes in the analysis of the last three interviews, suggesting saturation; we therefore conducted 20 interviews.

Literal transcriptions of selected relevant answers are shown in Additional Files 1, 2, 3, 4, 5 (Box A-E). In patient coding, F stands for female and M for male, followed by the age of patient. "Ehrm" and "Er" are formulas used to express doubt, or hesitation.

## Results

Twenty-three consecutive teenagers were approached and 20 agreed to be interviewed between January and April 2011 (10 males; average age 15.1 years). Two declined because of time constraints and one on the grounds that he "didn't think it would be fun". All but two participants were interviewed alone, the exceptions being for medical reasons. The allergic characteristics of patients are summarised in Table 1.

**Table 1:** Clinical characteristics of participants

Gender	Age (Yrs.)	Allergies	Prescribed auto- injector (years)	Ever used?	Severity of worst reaction	Age at 1^st ^reaction (Yrs.)
F	16	Peanut, soya	9	No	Mild/moderate	1
M	13	Peanut, brazil nut	8	No	Severe	5
M	12	Wasp sting	1	No	Mild	11
M	15	Egg	7	No	Severe	< 1
M	12	Cashew nut, hazelnut, strawberry	2	No	Mild	5
F	16	Fish	10	No	Moderate	< 1
F	16	Peanut, brazil nut, hazelnut, pecan nut	10	Yes	Severe	unknown
M	16	Hornet sting	2	No	Mild/moderate	15
M	14	Peanut, egg	10	No	Moderate	< 1
M	18	Brazil nut, grass, tree pollen	2	No	Severe	16
M	12	Peanut	5	No	Moderate/severe	7
F	16	Peanut, egg, dust mites	14	No	Severe	1
F	17	Peanut, hazelnut, almonds	3	No	Mild	unknown
F	12	Peanut, sesame	7	No	Mild	3
F	18	Wasp sting	4	No	Mild	14
F	13	Peanut, brazil nut, cashew	7	No	Severe	7
M	12	Milk, egg	8	No	Severe	< 1
F	16	All tree nuts except cashews	6	Yes	Severe	< 1
M	15	Horses, dogs	< 1	No	Severe	2
F	16	All nuts and peanuts	unknown	No	Severe	unknown

Six themes relating to whether teenagers carried their auto-injector were identified: role of circumstances, the type of allergy, factors associated with device design, the responsibility and attitude of others, and the teenager's feelings and concerns. Examples of quotes relating to each theme are in Additional Files (Boxes A-E). There were no notable differences in responses from patients who had previously suffered severe reactions in comparison to those who had experienced mild or moderate reactions.

### Role of Circumstances

All teenagers carried their auto-injector at least some of the time, although one only if he was visiting the hospital to 'keep the doctor happy' (Additional file 1, Box A;1). One participant said he always carried his auto-injector (Additional file 1, Box A;2), but most teenagers made decisions about whether to carry their medication, based on the situation they found, or expected to find themselves in. There were three elements of the context: the place, the people involved and the perceived likelihood of the presence of the allergen (Additional file 1, Box A).

The place could be evaluated in terms of familiarity, predictability and distance from auto-injector or external help. Going to a friends' house which was just next door was considered to be low risk (Additional File 1, Box A;3,4), and most considered keeping the auto-injector in the changing rooms whilst on the sports field acceptable, if they were food allergic and not planning to eat. Going on holiday, to a restaurant or somewhere unknown were considered high risk, and most would take their auto-injectors when going to these places. Almost all of the teenagers took their auto-injectors to school, although a few didn't, considering that it was familiar and predictable environment and that they felt in control. Social occasions like parties were a judgement call on the part of the teenager, who would weigh up the inconvenience of carrying the auto-injector against the unpredictable nature of the event.

The teenagers also assessed the situation in relation to who else was likely to be present (discussed in 'Responsibility of others' section). Where the adult in charge was not the legal guardian of the teenager, such as friends parents and teachers, some teenagers said they would be more likely to carry it.

The perceived likelihood of the presence of the allergen was also very important, for example, food allergic teenagers reported not worrying about going places away from home if they were not expecting to eat anything, for example walking the dog, or eating at a friends' house who knew to keep the allergen away from them (Additional File 1, Box A;5,6).

### Type of allergy

Food allergic teenagers had very different worries to venom allergic teenagers (Additional File 2; Box B). Individuals with food allergy were able to exert some control by deciding whether to eat. Several participants described a 'hierarchy' of food allergies, with peanut allergy seen as the worst to have, and individuals with allergies to fish and soya considering themselves at low risk in comparison (Additional File 2, Box B;1,2). Individuals with venom allergy have no such control, although the risk associated with venom allergy is seasonal, and the teenagers did not consider themselves at much risk in the winter (Additional File 2, Box B;3). Teenagers with venom allergies were more wary of certain locations (Additional File 2, Box B;4), and were vigilant about taking their auto-injectors to rural locations.

### Attitudes about the device

Features of the device itself significantly impacted on its carriage and acceptability for use (Additional File 3, Box C). The physical features were the main consideration and a large proportion of the teenagers commented that the size of the device influenced their decision not to carry it all of the time. The fact that the auto-injector is too big to be carried in a pocket recurred as a major issue, with boys mentioning it more than girls (Additional File 3, Box C;1). The needle and injection aspect of using the auto-injector worried several of the teenagers, one of whom was so scared of it, that she said she wouldn't use it even if she was having a severe reaction (Additional File 3, Box C;2). However, although fear of needles may impact on actual usage in the event of a reaction, there was no evidence from this study that it affected carriage of devices.

Some teenagers suggested preferable alternatives to injections such as an oral medication (Additional File 3, Box C;3). The packaging was considered alarming and scary by some, and the cardboard packaging was reportedly too flimsy and easily battered when kept in a bag. Almost all teenagers mentioned that it was irritating having to remember to take the auto-injector everywhere with them and it would be easier not to have to carry it. Some teenagers also mentioned other features of the device, such as the expiry dates and the temperature liability as extra things to worry about.

The teenagers' memories of their education regarding their allergy and auto-injector affected how they thought about and managed their situation. When asked to demonstrate how to use the auto-injector, a minority of the teenagers were unsure of how to do it in practise, despite having received training from an allergy nurse (Additional File 3, Box C;4,5). In contrast, many of the teenagers easily remembered being taught in the clinic how to use it, some regularly practised how to use them, involving their family, and one had taught all of their friends how to use it (Additional File 3, Box C;6). Most of the teenagers knew what to expect from a reaction, and what symptoms would prompt them to take either anti-histamines or use the auto-injector.

### Responsibility and attitudes of others

Whilst some of the teenagers managed their auto-injectors relatively independently, many relied on other people to shoulder the responsibility of carrying the auto-injector, ensuring it was in date and administering it if needed (Additional File 4, Box D). Independence did improve with age, and boys were more reliant than girls. The person most relied on was the mother (Additional File 4, Box D;1,2,3) but also the school nurse, father and teachers. When someone else was in charge of an aspect of managing the allergy, some mentioned that they didn't have to think about their allergy, so felt less concerned.

The attitudes of friends influenced the way teenagers perceived and managed their allergy. A few teenagers reported that members of their family or friends also had allergies, and this made it more acceptable for them (Additional File 4, Box D;4). Others had friends who had no understanding of allergies, who saw the auto-injector as something to make fun of, play around with or even steal (Additional File 4, Box D;5,6).

Positive reactions by others tended to increase the acceptance of their auto-injector, e.g. others learning how to use the auto-injector, being aware of their allergy, and not making a big deal out of it made the teenagers feel more comfortable with carrying their auto-injector (Additional File 4, Box D;7).

### Feelings and attitudes of allergic teenagers to auto-injectors

The way the teenagers felt about their auto-injectors was influenced by the interactions of factors from the previously mentioned themes. For example, a teenager whose parents carry the auto-injector for them most of the time would be less bothered by the size or shape of it, but may be less sure how to use it.

Some teenagers' concerns about the dangers of allergy manifest as stronger adherence to medical advice which created a sense of safety (Additional File 5, Box E;1). Other emotions could prevent carriage, for example embarrassment or "it won't happen to me" type-invincibility (Additional File 5, Box E;2,3). Some participants balanced their negative feelings against the perceived advantages of having the correct treatment (Additional File 5, Box E;4,5). For others the auto-injector was not considered a problem, just a normal part of life (Additional File 5, Box E;6).

Many teenagers commented that it was irritating to have to carry their auto-injector everywhere, but this did not affect how much they actually carried it. Most were aware that it was given to them for a very good reason, it was their 'lifeline' and although it was a pain, there wasn't much that could be done about it (Additional File 5, Box E;5.6). Some teenagers felt embarrassed by having to carry it, or did not want any extra attention because of it, but were able to deal with these emotions and did not let them affect their actions.

## Discussion

This is the first study to investigate factors and feelings that may inhibit or improve the carriage of epinephrine auto-injectors by teenagers under the care of a specialist allergy clinic. Although teenagers with food or venom allergy are generally in excellent health, the constant need for avoidance of allergen exposure and need to carry rescue medication impact on quality of life [17]. It was notable that the majority of teenagers wanted to remain safe and made risk assessments taking into account a number of factors including the likelihood of contact with an allergen and who else would be present to assist them should they have a reaction.

Our main finding was that adolescents are not making decisions with respect to their auto-injector that are especially irrational or hard to understand. They do however vary considerably in their approach to risk assessment and their auto-injector and live in a state of considerable uncertainty. More education about risk and risk assessment might be empowering for this group. A second key finding was adolescents' dissatisfaction with design of the currently available device, and some decisions not to carry the auto-injector were based on the physical design of the auto-injector and the hassles of carrying it, rather than informed risk assessments. Thirdly, most decision making was rational, although some decision making was based on rules of thumb that they had derived themselves rather than through medical advice or other reputable forms of information.

All participants were under specialist allergy services. It is our usual management that in addition to a medical and, where appropriate, dietetic review, the parents of younger children have one-to-one auto-injector counselling and training from an allergy nurse, are provided with personal written management plans and have leaflets concerning allergen avoidance and auto-injectors. Prior to secondary education we aim to repeat this education package for the child, almost invariably with the parent present. All participants had received such training. Through the teenage years we aim to gradually transition the patient to management independent of their parents [10].

We have previously shown that teenagers take risks when managing their allergies and a number of surveys have also shown that teenagers do not carry their auto-injectors at all times and eat foods that incur risks (5-9). Only one of the participants in the study reported carrying their auto-injector at all times; most would continually assess the possible risk of exposure to the allergen and whether or not they felt the need to carry the device. It is unlikely that any educational package could achieve 100% carriage of auto-injectors by teenagers and a more pragmatic approach is to empower them to make safe risk assessments eg. knowledge of the overall level of risk for mild reactions/severe reactions/fatal anaphylaxis in adolescents with food allergies, and an understanding of personal risk factors which might impact on this.

Although some of the teenagers expressed feelings about their allergies such as fear or anxiety, this did not necessarily improve adherence to carry their auto-injector. This finding supports a previous study on children and mothers in our clinic, which found that anxiety did not improve adherence to medical advice [8]. However in some cases the anxiety did appear to promote carriage and as noted in previous studies, the burden of allergy in teenagers seems to be relieved by having an auto-injector prescribed [8,18].

We found that many younger teenagers relied on their parents or teachers to take care of them should they have a reaction but this reliance on others appeared to diminish with increasing age. This is aligned with the educational programme of gradual transition of care in our clinic, in which the parents are initially totally responsible for the allergen avoidance and medical care of a very young child, with increasing independence of the child throughout childhood to a state in which they should be fully independent by the time that they transfer to adult services. However parents continue to provide support even for older teenagers and this might improve adherence with carriage and treating reactions [19]. It is noteworthy that all of the teenagers in the study were accompanied to the hospital by at least one parent.

Some of the participants in the study reported that their peers were well informed about their allergy and knew what to do should they have a reaction. These individuals seemed calmer about the prospect of a reaction. In peer-led asthma education programmes for adolescents participants have reported feeling more comfortable with their condition when their friends understood it [20].

Only two of the participants in the study had ever personally used their auto-injector although a number had been injected with epinephrine by a health professional. We ensure that all teenagers in the clinic have the opportunity to practice using a trainer auto-injector and also aim for them all at some stage during their time in our clinic to have used a 'live' auto-injector into a fruit or vegetable. Not all participants in this study had had the opportunity to use a 'live' auto-injector. It is clearly paramount that not only is the auto-injector carried but it is used appropriately either by the allergic individual themselves or by one of their friends and peer education is therefore of vital importance [13].

A major factor cited by most teenagers was the size and shape of their auto-injector and the presence of a needle was also predominant in some patients' decision as to whether they should use it. The auto-injector can only be used if it is being carried and it is vital that consideration is given to designing devices that are more acceptable and practical to be carried [21]. The packaging used to protect devices in situations where it is carried in a bag needs addressing to ensure that it is robust. Patients should be provided with information for them to obtain the hard cases that are commercially available.

Our findings compliment a primary care study by Gallagher et al [13] which investigated teenagers' attitudes and knowledge about their auto-injector, allowing comparisons between respondents who have received different allergy care packages. Our study has very different findings, presumably reflecting that our participants were under the care of a specialist centre and had received much of the education proposed by the previous study. Despite most of them having an acceptable level of knowledge regarding device usage, most described a physical and psychological barriers to carriage that need addressing.

## Conclusions and Clinical Implications

Our study demonstrates that the reasons for teenagers not carrying or using their epinephrine auto-injectors are multifaceted and complex. Despite being under the care of a specialist allergy clinic and having undergone a longitudinal educational programme, a number of decisions made by teenagers were based on the physical design of the device and the hassles of carrying it, rather than informed risk assessments. It is noteworthy that the majority of teenagers wanted to remain safe and generally made rational risk assessments. All of our teenagers have been trained in the use of epinephrine auto-injectors but unless they are actually carrying them at the time of a reaction, the risk of fatality increases. Our study highlights the need for individualised discussions regarding risk management. A single educational intervention is unlikely to work since the beliefs, emotions and lifestyle of each teenager is different and this impacted on their carriage and likelihood of using their auto-injector. We call on product designers and pharmaceutical companies to work on developing devices that are more acceptable to our patients and easier to carry, and from health care professionals to provide individualised educational packages for allergic teenagers.

## Competing interests

CM, JB, GS, M E-L, JH and JSL have nothing to declare. Graham Roberts is on the ALK-Abello advisory board but does not personally benefit financially from this activity; ALK-Abello have provided comsumables for his research studies. RK has acted as an advisor to Meda but has received no personal benefit.

## Authors' contributions

JSL was principal investigator with primary responsibility for the study and analysis of data. CM conducted all interviews, drafted the first manuscript and primary data analysis. JB provided expertise in qualitative interviews and qualitative data analysis. MEL, GS, RK and GR provided access to their clinics. JH provided advice from a patient perspective. All authors have participated in the design of the study and the preparation of the manuscript. All authors have seen and approved the final manuscript.

## Supplementary Material

Additional file 1 **Box A**. Quotes from participants. **Legend for Boxes: **Quotes are labelled as sex and age in years. Gender M = male; F = female. Direct quotes from participants are included. "Ehrm" and "Er" are formulas used to express doubt, or hesitation. Where a commercial name of a device was used the text has been amended to "auto-injector". Click here for file

Additional file 2 **Box B**. Quotes from participants. **Legend for Boxes: **Quotes are labelled as sex and age in years. Gender M = male; F = female. Direct quotes from participants are included. "Ehrm" and "Er" are formulas used to express doubt, or hesitation. Where a commercial name of a device was used the text has been amended to "auto-injector". Click here for file

Additional file 3 **Box C**. Quotes from participants. **Legend for Boxes: **Quotes are labelled as sex and age in years. Gender M = male; F = female. Direct quotes from participants are included. "Ehrm" and "Er" are formulas used to express doubt, or hesitation. Where a commercial name of a device was used the text has been amended to "auto-injector". Click here for file

Additional file 4 **Box D**. Quotes from participants. **Legend for Boxes: **Quotes are labelled as sex and age in years. Gender M = male; F = female. Direct quotes from participants are included. "Ehrm" and "Er" are formulas used to express doubt, or hesitation. Where a commercial name of a device was used the text has been amended to "auto-injector". Click here for file

Additional file 5 **Box E**. Quotes from participants. **Legend for Boxes: **Quotes are labelled as sex and age in years. Gender M = male; F = female. Direct quotes from participants are included. "Ehrm" and "Er" are formulas used to express doubt, or hesitation. Where a commercial name of a device was used the text has been amended to "auto-injector". Click here for file

## References

[B1] Gupta R Sheikh A Strachan DP Anderson HR Time trends in allergic disorders in the UK Thorax 2007 62 91 96 10.1136/thx.2004.038844 16950836 PMC2111268

[B2] Sampson MA Munoz-Furlong A Sicherer SH Risk-taking and coping strategies of adolescents and young adults with food allergy J Allergy Clin Immunol 2006 117 1440 1445 10.1016/j.jaci.2006.03.009 16751011

[B3] Rudders SA Banerji A Vassallo MF Clark S Camargo CA Jr Trends in pediatric emergency department visits for food-induced anaphylaxis J Allergy Clin Immunol 2010 126 385 388 10.1016/j.jaci.2010.05.018 20621344

[B4] Pumphrey RS Gowland MH Further fatal allergic reactions to food in the United Kingdom, 1999-2006 J Allergy Clin Immunol 2007 119 1018 1019 10.1016/j.jaci.2007.01.021 17349682

[B5] Simons FE Gu X Simons KJ Epinephrine absorption in adults: intramuscular versus subcutaneous injection J Allergy Clin Immunol 2001 108 871 873 10.1067/mai.2001.119409 11692118

[B6] Barnett J Leftwich J Muncer K Grimshaw K Shepherd R Raats MM Gowland MH Lucas JS How do peanut and nut-allergic consumers use information on the packaging to avoid allergens? Allergy 2011 66 969 978 10.1111/j.1398-9995.2011.02563.x 21320134

[B7] Barnett J Muncer K Leftwich J Shepherd R Raats MM Gowland Lucas JS Using 'may contain' labelling to inform food choice: a qualitative study of nut allergic consumers BMC Public Health 2011 11 734 10.1186/1471-2458-11-734 21943285 PMC3195759

[B8] Cummings AJ Knibb RC Erlewyn-Lajeunesse M King RM Roberts G Lucas JS Management of nut allergy influences quality of life and anxiety in children and their mothers Pediatr Allergy Immunol 2010 10.1111/j.1399-3038.2009.00975.x 20088863

[B9] Leftwich J Barnett J Muncer K Shepherd R Raats MM Gowland MH Lucas JS The challenges for nut-allergic consumers of eating out Clin Exp Allergy 2011 41 243 249 10.1111/j.1365-2222.2010.03649.x 21121977

[B10] Monks H Gowland MH MacKenzie H Erlewyn-Lajeunesse M King R Lucas JS Roberts G How do teenagers manage their food allergies? Clin Exp Allergy 2010 40 1533 1540 10.1111/j.1365-2222.2010.03586.x 20682004

[B11] Bock SA Munoz-Furlong A Sampson HA Fatalities due to anaphylactic reactions to foods J Allergy Clin Immunol 2001 107 191 193 10.1067/mai.2001.112031 11150011

[B12] Gallagher M Worth A Sheikh A Clinical allergy has much to gain from engagement with qualitative research Allergy 2009 64 1117 1119 10.1111/j.1398-9995.2009.02065.x 19432935

[B13] Gallagher M Worth A Cunningham-Burley S Sheikh A Epinephrine auto-injector use in adolescents at risk of anaphylaxis: a qualitative study in Scotland, UK Clin Exp Allergy 2011 41 869 877 10.1111/j.1365-2222.2011.03743.x 21481022

[B14] Muraro A Roberts G Clark A Eigenmann PA Halken S Lack G Moneret-Vautrin A Niggemann B Rance F The management of anaphylaxis in childhood: position paper of the European academy of allergology and clinical immunology Allergy 2007 62 857 871 10.1111/j.1398-9995.2007.01421.x 17590200

[B15] Hourihane JO Kilburn SA Dean P Warner JO Clinical characteristics of peanut allergy Clin Exp Allergy 1997 27 634 639 10.1111/j.1365-2222.1997.tb01190.x 9208183

[B16] Braun V Clarke V Using thematic analysis in psychology Qualitative Research in Psychology 2010 3 77 101

[B17] Cummings AJ Knibb RC King RM Lucas JS The psychosocial impact of food allergy and food hypersensitivity in children, adolescents and their families: a review Allergy 2010 10.1111/j.1398-9995.2010.02342.x 20180792

[B18] MacKenzie H Roberts G van LD Dean T Teenagers' experiences of living with food hypersensitivity: a qualitative study Pediatr Allergy Immunol 2010 21 595 602 19702674 10.1111/j.1399-3038.2009.00938.x

[B19] Kim JS Sinacore JM Pongracic JA Parental use of EpiPen for children with food allergies J Allergy Clin Immunol 2005 116 164 168 10.1016/j.jaci.2005.03.039 15990790

[B20] Gibson PG Shah S Mamoon HA Peer-led asthma education for adolescents: impact evaluation J Adolesc Health 1998 22 66 72 10.1016/S1054-139X(97)00203-6 9436069

[B21] Frew AJ What are the 'ideal' features of an adrenaline (epinephrine) auto-injector in the treatment of anaphylaxis? Allergy 2011 66 15 24 10.1111/j.1398-9995.2010.02450.x 20716315

