# Supplementary material for: What factors affect the carriage of epinephrine auto-injectors by teenagers?
Source: Clin Transl Allergy. 2012 Feb 2;2:3. doi: 10.1186/2045-7022-2-3 (PMC3299626; doi:10.1186/2045-7022-2-3)
Supplement: Additional file 1 — Box A. Quotes from participants. Legend for Boxes: Quotes are labelled as sex and age in years. Gender M = male; F = female. Direct quotes from participants are included. "Ehrm" and "Er" are formulas used to express doubt, or hesitation. Where a commercial name of a device was used the text has been amended to "auto-injector". [file 2045-7022-2-3-S1.DOC]

| **Box A. Role of circumstances.** | |
| --- | --- |
| *1.*  *2*  *3*  *4*  *5*  *6* | *Researcher:* So do you have it with you now?  *M18:* Er I do, but that’s only because ehrm since this is my third year for Pollinex and er for some reason this year they’ve, er they’ve got a new consultant and he comes up for every, every time I have the treatment, before and after the checks it’s been okay, and he doesn’t like it if I don’t turn up without it, so I do it just to keep him happy really.  *M12:* And we’ve never really gone out anywhere else that’s not in school and not had it with us.  Researcher: Can you think of a time when you know you didn’t have it?  *M12*: Er ... ehrm well sometimes when I go around my friends’ houses, but they’re only kind of, well a short way from my house.  *F18:* Ehrm I’d probably sometimes forget if I just pop out of the house for half an hour  *M12:* Er well if I went out with friends, if we went out for a lunch or went out for dinner somewhere, or I went over to one my friends’ houses and had something to eat there, I would always bring it. I bring it to like, if I was on holiday I would bring it, because I need to take some other pens as well.  *F17:* Ehrm I’ve probably forgotten it more than once, just to, yeah say if I went out for a couple of hours and normally if I’m pretty sure I’m not gonna eat anything, I’m more likely to forget it! Ehrm I, I always make sure if I’m definitely eating, I definitely have it on me, but ehrm. |
