# Supplementary material for: What factors affect the carriage of epinephrine auto-injectors by teenagers?
Source: Clin Transl Allergy. 2012 Feb 2;2:3. doi: 10.1186/2045-7022-2-3 (PMC3299626; doi:10.1186/2045-7022-2-3)
Supplement: Additional file 2 — Box B. Quotes from participants. Legend for Boxes: Quotes are labelled as sex and age in years. Gender M = male; F = female. Direct quotes from participants are included. "Ehrm" and "Er" are formulas used to express doubt, or hesitation. Where a commercial name of a device was used the text has been amended to "auto-injector". [file 2045-7022-2-3-S2.DOC]

| **Box B. Type of allergy** | |
| --- | --- |
| *1*  *2*  *3*  *4*  *5* | *F16:* I feel I’ve been quite lucky to have a fish allergy, because I can kind of smell it, you know it’s not really a hidden ingredient like nuts are, ehrm so I’m just very thankful that I grew out of nuts.  *F18:* Ehrm well it’s a bit inconvenient, but not overly. I mean it’s not; it’s probably not as bad as having one which is more prevalent to like foods and that sort of thing.  *Researcher:* And how often do you carry the “auto-injector”?  *F18:* Not in winter as much, only when, unless I’m going somewhere where I expect wasps to be, but in the summer, yeah, mostly.  *M12:* With a wasp allergy you’ve got to be careful where you go you don’t walk into a bush where there’s a wasps’ nest or something.  *M12:* It makes you in a way feel paranoid when you go places. |
