# Supplementary material for: What factors affect the carriage of epinephrine auto-injectors by teenagers?
Source: Clin Transl Allergy. 2012 Feb 2;2:3. doi: 10.1186/2045-7022-2-3 (PMC3299626; doi:10.1186/2045-7022-2-3)
Supplement: Additional file 3 — Box C. Quotes from participants. Legend for Boxes: Quotes are labelled as sex and age in years. Gender M = male; F = female. Direct quotes from participants are included. "Ehrm" and "Er" are formulas used to express doubt, or hesitation. Where a commercial name of a device was used the text has been amended to "auto-injector". [file 2045-7022-2-3-S3.DOC]

| **Box C. Attitudes about the device** | |
| --- | --- |
| *1*  *2*  *3*  *4*  *5*  *6* | *M18:* I don’t think it’s an image thing……..I think it’s just basically their size. If they could make it quite a lot smaller, I think most people would just pop it in their pocket or in their wallet or with them, you know keep it on them.  *Researcher:* How bad do you think it would have to get for you to use it?  *F16*: Ehrm very bad probably, but I dunno, I’d probably have to be pinned down for them to use it on me because I don’t like it.  *M12:* Well I don’t know whether this is possible, but if you could make it so you could drink it, I’d prefer it, cause I don’t really like jabbing my leg, when you think you’re stabbing, you’re jabbing it into your leg and I have a fear of when the needle comes out and stabs you in the leg. I think I’d rather have something it’d be quite pleasant to have all the time.  *M14:* Oh God, I’ve forgot. I think you take that off, and then like you have to put it in there, and then like press it down and then count to, oh no, [laughs], I dunno. And then you have to count, and then like release it. Help!  *M12:* I’ve never actually used one. I’ve seen it being done, but……  *Researcher*: At the Allergy Clinic, have they told you anything about it?  *M12*: I’ve seen it, I’ve seen it being used on an onion  *M13:* (Family) Yeah, they all had to have like a practice session a few weeks ago, just to check that we all knew how to do it. |
