# Supplementary material for: What factors affect the carriage of epinephrine auto-injectors by teenagers?
Source: Clin Transl Allergy. 2012 Feb 2;2:3. doi: 10.1186/2045-7022-2-3 (PMC3299626; doi:10.1186/2045-7022-2-3)
Supplement: Additional file 4 — Box D. Quotes from participants. Legend for Boxes: Quotes are labelled as sex and age in years. Gender M = male; F = female. Direct quotes from participants are included. "Ehrm" and "Er" are formulas used to express doubt, or hesitation. Where a commercial name of a device was used the text has been amended to "auto-injector". [file 2045-7022-2-3-S4.DOC]

| **Box D: Responsibility and attitudes of others** | |
| --- | --- |
| *1*  *2*  *3*  *4*  *5*  *6*  7. | *M12:* Well my mum, she keeps them on the counter, so she always keeps an eye on them and makes sure they’re in date, and if they’re not, she’ll come and tell me.  *M15:* Ehrm well my mum always has one in her handbag when I’m with her, and my dad has one in the car, and there’s one at school in one my locker and the school matron has one.  *M12:* I’d probably get my mum to do it.  *F16*: Yeah, my, well my mum’s got an allergy herself, so most of my family will know what to do and ehrm yeah, yeah it’s fine.  *M12:* sometimes my friends take the Mick out of me for having all this medicine and stuff with me all the time.  *Researcher:* What’s the worst thing about having to carry an “auto-injector”?  *M18:* Ehrm having to carry the “auto-injector”, and having to explain to people, you know what the “auto-injector” is for, because they see it and they freak.  *F16*: Like all my friends know how to use it. Yeah, like ehrm they would like come round home, like a few of my friends, so we can use an out-of-date “auto-injector” on oranges and stuff like that. |
