# Supplementary material for: What factors affect the carriage of epinephrine auto-injectors by teenagers?
Source: Clin Transl Allergy. 2012 Feb 2;2:3. doi: 10.1186/2045-7022-2-3 (PMC3299626; doi:10.1186/2045-7022-2-3)
Supplement: Additional file 5 — Box E. Quotes from participants. Legend for Boxes: Quotes are labelled as sex and age in years. Gender M = male; F = female. Direct quotes from participants are included. "Ehrm" and "Er" are formulas used to express doubt, or hesitation. Where a commercial name of a device was used the text has been amended to "auto-injector". [file 2045-7022-2-3-S5.DOC]

| **Box E. Feelings and attitudes affecting carriage of auto-injectors** | |
| --- | --- |
| *1*  *2*  *3*  *4*  *5*  *6* | *M12:* I feel a lot safer when I’ve got it with me. If I don’t have it with me I start to worry a bit, but I get over it and when I have it with me I tend to have it on me, so it’s on a bag on me or I’m carrying it.  *M13:* Err if you’re the only one carrying an “auto-injector” in your bag, a very bulky bag, it’s not very comfortable and don’t want the attention on me really.  *Researcher:* When was the last time you took it out with you?  *M18:* Ehrm I can’t actually remember, so it was that long ago. I feel fine. I don’t feel like I’m gonna need it really, cause I’ve, I’ve, cause I’ve never had to use it, I’ve never had, since, since I had this problem.  *M12:*  If I was having an anaphylactic shock, I won’t really be concentrating on the needle will I?  *M12:* Bit of a pain sometimes, but you know if it keeps you healthy….  *M13*: I manage to live with it, I’ve lived with it, I’m fine with it. |
